# Evaluation of an AI-Based Chatbot Providing Real-Time Feedback in Communication Training for Mental Health Care Professionals: Proof-of-Concept Observational Study

**DOI:** 10.2196/82818

**Published:** 2025-11-28

**Authors:** Lea Herschbach, Teresa Festl-Wietek, Christian Stegemann-Philipps, Alessandra Sonanini, Bernd Herrmann, Rebecca Erschens, Anne Herrmann-Werner, Friederike Holderried

**Affiliations:** 1 TIME - Tuebingen Institute for Medical Education University of Tuebingen Tuebingen Germany; 2 Department of Psychosomatic Medicine and Psychotherapy University Hospital Tuebingen Tuebingen Germany

**Keywords:** artificial intelligence, clinical competence, communication, feedback, medical education, physician-patient relations, simulated patients

## Abstract

**Background:**

Effective communication is essential in medical practice, especially in dealing with the increasing number of patients presenting with mental health conditions. Feedback plays a crucial role in improving communication skills but is often difficult to implement. Artificial Intelligence (AI)–based tools offer promising support for practicing and improving communication techniques among physicians.

**Objective:**

This proof-of-concept study investigated the capability of an AI-based chatbot that provided physicians with real-time feedback to train communication techniques. Specifically, it addressed three research questions: (1) How accurate is the AI-generated feedback, and how is it perceived by participants? (2) Does the use of an AI-based chatbot and AI-generated feedback lead to an increased frequency in the use of specific communication techniques? (3) Do physicians perceive the training with the AI-based chatbot and AI-generated feedback as beneficial for their daily clinical practice?

**Methods:**

The study used a proof-of-concept design using 56 anonymized chat transcripts between physicians and simulated patients. Participating physicians received automated feedback generated by the AI-based chatbot, which was designed to assess and encourage the use of specific communication techniques. Feedback accuracy and user perception were evaluated, and changes in communication behavior were assessed. Finally, participants completed a postintervention questionnaire to evaluate perceived benefits for clinical practice.

**Results:**

The AI-generated feedback was found to be very accurate with 85.38% of the real-time feedback being partially or even totally correct. In addition, most of the participants considered the feedback accurate, with 83.64% (n=55) agreeing or totally agreeing. Furthermore, 87.27% (n=55) of the participants agreed or totally agreed to the fact that the feedback was helpful. It also appeared to support the repeated use of recommended communication techniques. Furthermore, most of the participants agreed that practicing with an AI-based chatbot helped them practice and apply new communication techniques in clinical interactions, improved their physician-patient communication, and helped them recognize mental health conditions better in everyday medical practice.

**Conclusions:**

The results highlight the potential of AI-supported training to address key communication challenges in medicine, particularly in the context of mental health care. The combination of real-time practice and immediate feedback may foster sustained improvements in physician-patient interactions. Integrating such tools into medical education could offer a valuable complement to traditional training methods. Future research may aim to refine AI models to improve their reliability and investigate the long-term effects and objective measures.

## Introduction

One of the core competencies of medical professionals is communication, with up to half of a physician's working time devoted to patient interactions [1,2]. Effective communication between physicians and patients can positively impact patient comprehension, cooperation, and trust, and therefore has a significant impact on the success of treatment [2,3]. Despite this, key communication skills are sometimes insufficiently represented in medical curricula [4,5]. Standardized patients offer realistic practice opportunities but are time and resource intensive, limiting scalability [6-8]. By using artificial intelligence (AI) to simulate patients, physicians can practice communication more flexibly and on a larger scale. Previous studies have demonstrated that AI-based simulations provide realistic patient interactions, offer subsequent feedback on history-taking, and generate mostly plausible responses [9,10]. AI is a valuable tool in medical education, offering accurate and automated assessments to enhance learning and skill development and improve patient outcomes [11-13]. However, effective integration requires maintaining students' independent and critical thinking, ensuring outputs are evidence-based and ethically sound. Accuracy, bias, privacy, and overreliance on technology must also be considered [12,14].

Feedback is essential to improve communication skills and for effective learning in clinical practice, but the type of feedback and the manner in which it is administered can have different effects [15,16]. In the context of communication, it is often challenging and costly to obtain timely and individualized feedback. Barriers such as inadequate training of supervisors, unfavorable learning environments, and lack of time make the feedback process more difficult [17]. AI models can be used to recognize and categorize communication techniques such as motivational interviewing (MI). MI is a patient-centered counseling approach that aims to enhance intrinsic motivation for behavior change through empathy, reflective listening, and guided exploration of ambivalence [18]. In addition to the categorization of communication techniques, these AI models can also deliver immediate, tailored feedback, supporting skill refinement during and after training without resource intensive supervision [19-21]. This feedback has been shown to assist participants in the refinement of their communication techniques during and following the training period [19,22].

Effective communication is crucial in the care of patients with mental health conditions, as these encounters often require heightened sensitivity, empathy, and adaptability [23-25]. The prevalence of mental illnesses is increasing worldwide [26,27], with up to 30% of outpatient and inpatient medical care allocated to the treatment of mental illnesses in Germany [2]. General practitioners (GPs) are typically the first point of contact for mental health problems [28,29]; yet, limited training and the high demands of diagnosis and treatment make the care of these patients particularly challenging [30-33].

Most studies on AI-generated feedback provide posttask rather than real-time feedback, despite a growing demand for dynamic systems that adapt feedback focus and complexity in real time [34]. One reason for this is technical complexity, since multiple distinct large language model (LLM) calls and a control flow to accommodate for the parallelized LLM responses are required. While machine learning–based real-time feedback has shown high usability and improved knowledge elaboration, interaction, and group performance [35,36], its application in the context of LLMs and communication remains largely untested.

In summary, prior research has shown the importance of communication in medicine and highlighted training gaps, particularly in interactions with patients with mental health disorders. However, there are few studies that examine the potential of real-time AI feedback to enhance communication techniques. The benefits of simulated patients and AI for communication skills have already been demonstrated but these have mostly focused on single techniques (like MI) and provided feedback after the interaction. The accuracy of AI-generated feedback for communication techniques beyond MI, its effectiveness in real-time application, and its long-term impact on skill retention have not been systematically studied. Addressing these gaps is essential to optimize AI-supported communication training and improve clinical outcomes, which is why we aim to examine the following questions in this study: (1) How accurate is the AI-generated, real-time feedback on communication techniques and how is it perceived by the participants? (2) Does the use of an AI-based chatbot and AI-generated feedback lead to an increased frequency in the use of specific communication techniques? (3) Do physicians perceive the training with the AI-based chatbot and AI-generated feedback as beneficial for their daily clinical practice?

The objective of this study is to test the hypothesis that an AI-based chatbot providing real-time feedback enhances physicians’ use of targeted communication techniques. Specifically, the study examines the accuracy, user perception, and impact of AI-generated real-time feedback on communication skills. The intervention is grounded in established frameworks of empathic and motivational communication, including Roger’s therapeutic stance, Linehan’s validation, and MI, which guide both the design of AI feedback and its application in clinical training.

## Methods

### Study Outline

This study was designed as a proof of concept to evaluate the feasibility and acceptability of real-time AI-generated feedback in communication training. Data collection took place between February and June 2025 as part of a training class in mental health for physicians at the University Hospital of Tübingen, Germany. Participants were divided into 2 groups to counterbalance case order, with group 1 starting with case A and group 2 with case B. After a thematic introduction and instructions on the communication techniques (see Table 1), participants conducted a 20-minute chat with an AI-simulated patient. They used provided laptops and received real-time feedback on the communication techniques used. This was followed by an assessment of the patient (diagnosis and recommended actions) and receiving overall feedback on communication technique application. The feedback summarized the techniques used, presented absolute and relative usage frequencies, highlighted underused techniques, and presented examples of their correct application. In addition, the completeness of the medical history was evaluated. Participants then rated whether this feedback was perceived as accurate and helpful, using a 5-point Likert scale from “totally disagree” to “totally agree.” The procedure was then repeated with a second AI-simulated patient chat, resulting in 2 chats per participant. After the interaction with the AI, a short survey was conducted, which collected demographic data and assessed the subjective perception of the tool’s authenticity on the same 5-point Likert scale described above. Furthermore, the following 4 questions were asked specifically: “I have the impression that practicing with an AI-supported chatbot can help me...” (1) “…practice new communication techniques,” (2) “…apply communication techniques in everyday clinical practice,” (3) “…better recognize a depression or anxiety disorder in everyday clinical practice,” and (4) “…improve physician-patient communication in everyday clinical practice.”

**Table 1:** Overview of communication techniques used with examples from the chats (translated to English).

Theory and technique		Explanation	Example
**Roger’s therapeutic stance**			
	Empathy	This refers to the therapist’s ability to understand the client’s experience without getting lost in those feelings.	GP^a^: “It’s always really unpleasant, this staying awake and digging and not being able to fall asleep. I understand that.”
**Motivational interviewing**			
	Open questions	Open questions invite others to tell “their story” in their own words without directing them in any particular direction.	GP: “What brings you to me?”
	Reflections	Rephrasing a statement to capture the implicit meaning and feeling of a patient’s statement.	P^b^: “My studies and my relationship suffer because I am constantly tired and irritable. This naturally leads to conflicts.” GP: “There is a lot of pressure on you to live up to yourself and your relationship.”
	Summarizing	Summarizing what has been said at a later stage, especially at transition points. Often end with an invitation or question.	GP: “I would like to summarize once again: you have been suffering from sleep problems, a loss of concentration and reduced energy in everyday life for half a year, is that correct?”
**Validation according to Linehan**			
	Verbalizing	Emotional reactions that are not expressed but are implied or conclusive from the behavior are validated.	GP: “I can see that you are very anxious; is that right?”
	Validating in terms of past experiences	Emotional reactions are related to the medical history or biographical or biological aspects and normalized as appropriate.	GP: “After such a bad experience, it is very understandable to be afraid of another attack.”
	Validating in terms of the current situation	Emotional reactions are related to the current situation and normalized as appropriate.	P: “But I’m still worried that it could be something serious” *looks anxious*. GP: “I understand that. IT’s really scary when you feel your heat racing and you start sweating.”
	Radical genuineness	The patient is seen as a partner on an eye-level relationship, targeting self-opening.	GP: “Yes, I can understand that, I also prefer not to be in crowded places, too much hustle and bustle.”
	Cheerleading	Believing in the patient is emphasized, positive characteristics of the patient are related to therapy.	GP: “Very good. I’m pleased that you want to get on board [with therapy].”

^a^GP: general practitioner.

^b^P: patient.

**Figure 1:**
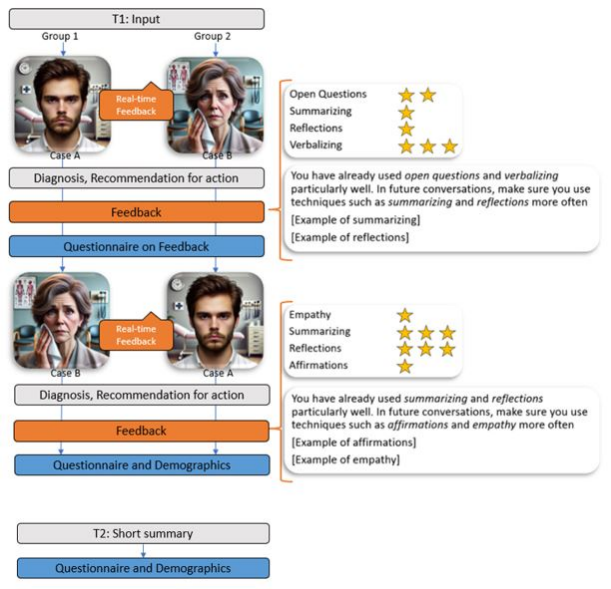
Outline of the experimental setup and the steps of data collection. T1: February 7, 2025; T2: June 27, 2025.

Participants answered the questions using the same 5-point Likert scale from “totally disagree” to “totally agree.” Additionally, a preliminary diagnosis had to be formulated. Electronic consent for anonymous data storage and analysis was obtained. Since data collection took place during a class and all participants were supposed to receive the same learning opportunities, a control group that did not practice with the tool was not included. Due to the lack of a control group and the small sample size, causal conclusions are limited. Nevertheless, the findings provide essential initial insights into the topic. A detailed flowchart illustrating the study procedure is presented in Figure 1.

To further explore the long-term implications of training with AI-simulated patients, a follow-up survey was administered to the same participants 5 months later. They answered the same 4 questions mentioned above, with their responses this time reflecting their experiences during the preceding months.

### Material

#### AI-Simulated Patients

We used a GPT-4 based chatbot with a custom interface, which allows us to enter individual patient vignettes, constantly manipulate chat history, and use elaborate prompting. It enabled students to submit written questions to a virtual patient, receive responses, and save the complete chat history for further analysis. This application is described in detail in a previous studies [10,37].

In this study, 2 patient cases were developed by experts. The first case depicts a 28-year-old man with depression; the second case portrays a 46-year-old woman with agoraphobia and panic disorder. The cases are based on the *DSM-5* (*Diagnostic and Statistical Manual of Mental Disorders* [Fifth Edition]) [38] diagnostic criteria and were selected based on the prevalence of the disorders. The cases were integrated into the chatbot and comprehensively tested.

#### Communication Techniques

A literature review identified validated techniques for conducting empathic dialogue. Three well-established frameworks were selected, Roger´s therapeutic stance [39], validation according to Linehan [40] and MI [18], each of which provides a validated framework for core communication techniques. These frameworks guided the design of the AI-based training and the feedback provided to participants. Items not applicable to text-based chats (eg, “Unconditional positive appreciation” and “Listening and observing”) were not used. Similar or overlapping techniques (eg, “Congruence” and “Radical Genuineness,” “Affirmations” and “Cheerleading,” and “Reflections” from MI and validation according to Linehan) were merged. The selected techniques are summarized in Table 1. To prepare a training dataset, 2 experts independently evaluated 7 chats conducted for training purposes that asses which communication techniques were used and when. We did this to check how accurate we can expect AI to be when even human experts do not always agree. Initial coding yielded moderate agreement (κ=0.426, *P*<.001) due to the complexity of the material and slight differences in interpretation. After discussion and refinement, coding was repeated, resulting in good agreement (κ=0.689, *P*<.001). This evaluated training dataset was then integrated into the AI tool so that the feedback rules could be trained.

#### Feedback

The study incorporated various effective feedback types [41,42]. Formative feedback was provided immediately within the chat flow, specifying which communication technique had been applied, thereby confirming its use and encouraging further application. Summative feedback was visualized as a star-based graph showing the frequency of each technique after the chat. Future-oriented feedback highlighted the 2 least frequently applied techniques, illustrating them with examples to support their increased use in future conversations. A visual example of the feedback presented can be found in Multimedia Appendix 1.

#### Training the AI

The training dataset was integrated into the application to enable AI-generated feedback during data collection. An initial *F*_1_-score of 0.38 revealed frequent false positives, mainly due to the low frequency of certain techniques in the training data. The *F*_1_-score is the harmonic mean of precision and recall [43]. To improve accuracy, the definitions of the communication techniques [44] were refined and supplemented with positive and negative examples in the prompt. This raised the *F*_1_-score to 0.58, which was deemed sufficient. In this training context, a slightly higher rate of false positives (ie, techniques detected that were not present) was considered didactical acceptable, even beneficial, because it encourages participants to critically engage with their communication and reflect on technique use.

#### Prompt Development

The basic prompt structure for the chats was already available from the AI-based patient model that was used [10]. For the real-time feedback version gpt-4o-2024-08-06 was used (Multimedia Appendix 2). Prompts for generating feedback on communication techniques were refined in a manual, iterative process, continuously evaluating error rates for all items and reviewing cases where the AI’s feedback differed from our hand annotated data. Most of the major improvements resulted either from providing examples or from changing the specific wording. Wording was iteratively changed to make the prompts more precise and to make distinctions between the categories clearer. The exact prompts can be found in Multimedia Appendix 3. To implement the real-time feedback, multiple, distinct LLM-calls were necessary to avoid overloading one single prompt and to be able to choose appropriate models for each task.

### Data Analysis

All data were collected anonymously through the AI application. Participants’ ratings of feedback helpfulness and accuracy, as well as communication performance measures, were automatically recorded. After data collection, 56 chats from 28 participants were exported and included in the analysis, with 1 participant completing the chats but not all of the questionnaires. In the follow-up survey, 26 physicians of the same cohort participated. Chats were segmented into question-answer pairs (QAPs), with each participant’s question paired with the subsequent AI-generated response. They then were aggregated into a Microsoft Excel 2408 spreadsheet. The questionnaires were analyzed descriptively regarding demographics and performance. Statistical analyses were conducted in R (version 4.4.1; R Core Team, 2024) and SPSS (version 30; IBM Corp, 2023). Statistical significance was set at *P*<.05. Means, SDs, percentages, Pearson correlations, and *F*_1_-scores were calculated. To examine the effectiveness of AI-generated feedback in promoting the use of specific techniques, the frequency of technique usage in chat 1 was compared to usage counts in chat 2 by calculating Poisson regressions. For each participant, the 2 techniques explicitly mentioned in the feedback from the first chat, including examples, were selected for analysis. Figures were created using Excel 2408. As the evaluation required specialized domain knowledge and consistency, one expert reviewed all chats and rated the AI-generated feedback as (partially) correct or incorrect. Given that all feedback originated from the AI system, the evaluation was not blinded to this fact.

### Ethical Considerations

The study was approved by the Ethics Committee of the Faculty of Medicine at University Hospital Tübingen (597/2024BO2). All participants provided informed consent. Privacy and confidentiality were safeguarded through deidentification and secure data management practices. Data were processed anonymously and no conclusions could be drawn about participants. Participation in the study was voluntary and no form of compensation was provided.

## Results

### Demographics

In the first data collection, 18 participants identified themselves as female and 9 as male. The average age was 38.2 (SD 11.1) years. Ten participants worked in a hospital, 15 were in private practice, 2 stated other working fields. Thirteen participants were in the field of general medicine, 4 were in internal medicine, 3 were in gynecology, 2 were in neurology, 1 was in pediatrics, and 4 was in other fields. On average, they had 10.30 (SD 8.55) years of professional experience. A total of 56 chats were evaluated. On average, a chat consists of 19.19 (SD 6.46) QAPs. A total of 1108 QAPs were included in the evaluation. In the follow-up survey, 18 participants identified themselves as female and 8 as male. The average age was 40.88 (SD 8.19) years, the professional experience was 11.15 (SD 8.80) years. Sixteen participants were in private practice, 9 worked in a hospital, and 1 stated “other setting.” In total, 14 participants were in the field of general medicine, 3 were in gynecology, and 2 each were in neurology and in pediatrics and internal medicine. Three participants stated being in other fields.

### Correctness of the Diagnosis

Of the 28 participants, 17 (60.71%) correctly diagnosed both patients, 8 (28.57%) correctly diagnosed one, and 3 (10.71%) did not diagnose any patient correctly.

### Accuracy of Feedback

Expert evaluation of the AI-generated feedback demonstrated high overall accuracy with 876 out of 1108 (79.06%) QAPs identified completely correct. Seventy (6.32%) QAPs were identified as partially correct, meaning at least one of several feedbacked techniques was correct. A total of 81 (7.31%) QAPs were categorized as false positive and 70 (6.32%) QAPs as false negative. In 11 QAPs (1.00%), the techniques mentioned in the feedback were both false positives and false negatives (Multimedia Appendix 4). The AI rating was assessed as false positive in 4 out of 10 (40.00%) cases for “including past experiences,” in 13 out of 41 (31.71%) cases for “including current situation” and in 88 out of 277 (31.18%) cases for “open questions.” False negatives occurred most frequently for “including past experiences” (6/10, 60.00% cases), “including current situation” (17/41, 41.46% cases), and “empathy” (30/79, 37.97% cases).

**Figure 2:**
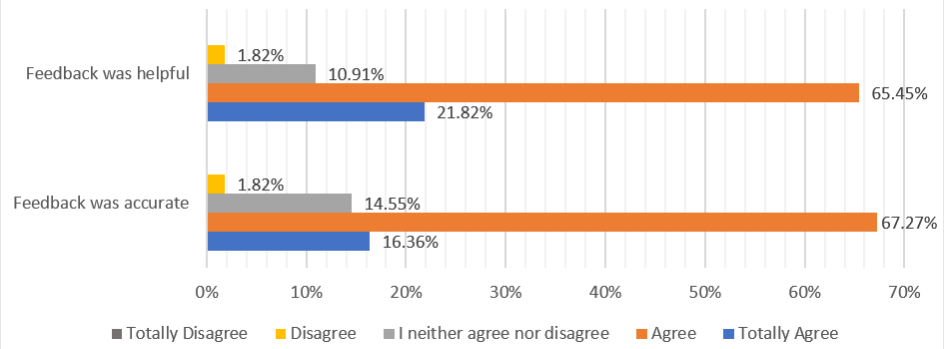
Participants´ assessment of the AI-generated feedback. AI: artificial intelligence.

These findings align with the participants’ perceptions. Across all 55 responses (28 participants completing 2 rounds each), 83.64% (n=55) agreed or strongly agreed that the feedback was accurate. Similarly, 87.27% (n=55) agreed or totally agreed to the fact that the feedback was helpful, with just one disagreement (1.79%, n=55; Figure 2).

### The Use of Specific Communication Techniques

**Figure 3:**
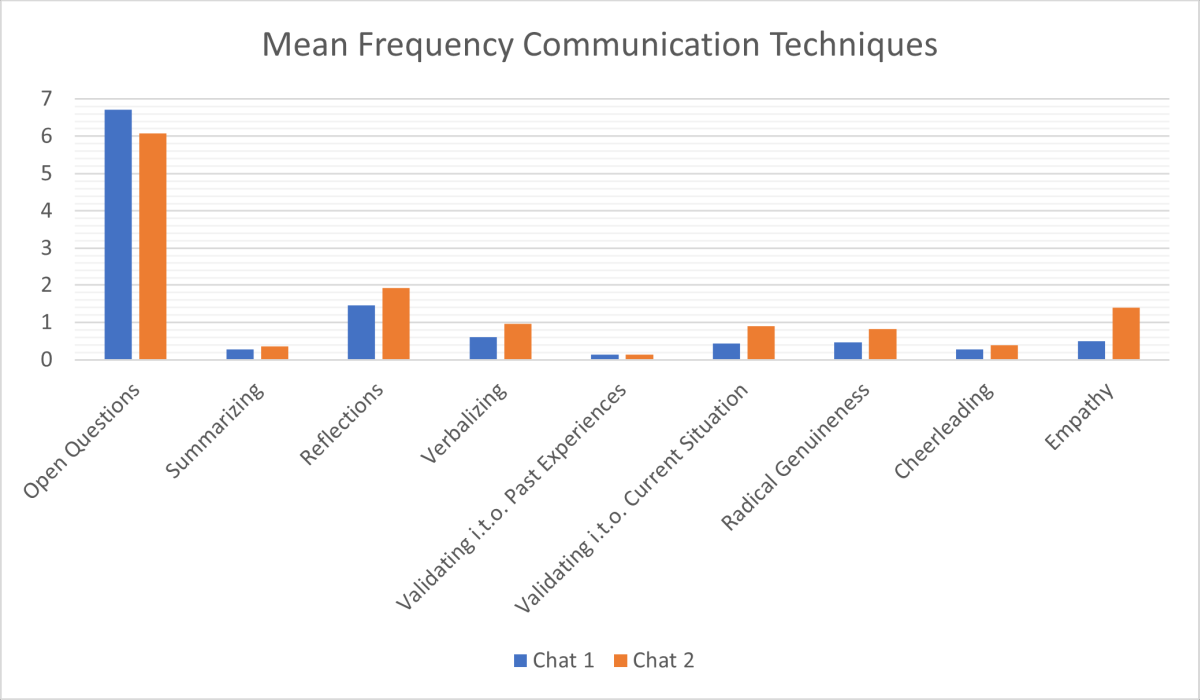
The mean frequency of communication technique usage in chat 1 and chat 2. i.t.o.: in terms of.

A Poisson regression was conducted to model the number of technique occurrences in the second chat as a function of prior feedback. Model criticism showed that the Poisson regression is properly modeling the data. The analysis revealed a significant increase in technique usage after receiving AI-generated feedback (β=1.365, SE 0.323; *z*=4.221; *P*<.001). The estimated mean count of techniques (*λ*) was 0.21 in chat 1 and 0.84 for chat 2. These findings suggest that AI-generated feedback effectively reinforces the use of recommended techniques in subsequent conversations (Figure 3).

### Subjective Benefits in Clinical Practice

In the next step, we examined the extent to which the physicians perceived practicing with the AI-generated patients as subjectively beneficial for their daily clinical work. Overall, 85.19% (n=27) of the participants agreed or totally agreed to the statement that practicing with an AI-supported chatbot can help them to practice new communication techniques and to apply them in everyday clinical practice (85.19%, n=27 agree or totally agree). Furthermore, 81.48% (n=27) agreed or totally agreed that practicing could help them to improve their physician-patient communication in their daily clinical work. Furthermore, the majority indicated that it may help them to better recognize depression and anxiety disorders in everyday clinical practice, with 59.26% (n=27) agreeing or totally agreeing. The exact agreement rates are shown in Table 2. These results were replicated in the follow-up survey 5 months later. Again, the majority of the 26 participants stated that, according to their subjective perception, practicing with the AI-supported chatbot enabled them to practice new communication techniques, apply them in everyday clinical practice and improve their physician-patient communication. They also reported that, in their own perception, they were better able to recognize depression and anxiety in their clinical practice after the training (Table 2).

**Table 2:** The participants’ assessments of the benefits of the application in everyday clinical practice at measurement time point 1 (February 2025, N=27) and measurement time point 2 (June 2025, N=26).

Response to “I have the impression that practicing with an AI-generated Chatbot can…” and time point		Totally agree, n (%)	Agree, n (%)	I neither agree nor disagree, n (%)	Disagree, n (%)	Totally disagree, n (%)
…**help me practice new communication techniques.**						
	T1	3 (11.11)	20 (74.07)	3 (11.11)	1 (3.70)	0 (0)
	T2	0 (0)	16 (61.54)	7 (26.92)	3 (11.54)	0 (0)
…**help me apply communication techniques in everyday clinical practice.**						
	T1	5 (18.52)	18 (66.67)	3 (11.11)	1 (3.70)	0 (0)
	T2	2 (7.69)	14 (53.85)	9 (34.62)	1 (3.85)	0 (0)
…**help me better recognize a depression or anxiety disorder in everyday clinical practice.**						
	T1	1 (3.70)	15 (55.56)	7 (25.93)	3 (11.11)	1 (3.70)
	T2	1 (3.85)	14 (53.85)	8 (30.77)	3 (11.54)	0 (0)
…**help me improve physician-patient communication in everyday clinical practice.**						
	T1	2 (7.41)	20 (74.07)	3 (11.11)	2 (7.41)	0 (0)
	T2	0 (0)	12 (46.15)	13 (0.50)	1 (3.85)	0 (0)

### Authenticity

Most of the participants (n=27, 62.96%) agreed that the AI-simulated patients appeared authentic, and an additional 11.11% (n=27) strongly agreed. Only 7.41% (n=27) disagreed; no one strongly disagreed. Pearson correlation analysis revealed a strong positive relationship between perceived authenticity and overall agreement with the chatbot’s usefulness (*r*_28_=0.696, *P*<.001), suggesting that higher perceived authenticity is associated with greater perceived benefit.

## Discussion

### Principal Findings

Our findings indicate that AI-generated real-time feedback is mostly accurate and is also perceived as such by the participants. This finding demonstrates the capability of AI technologies for generating prompt feedback on communication techniques. Furthermore, training with the AI-based chatbot with real-time feedback led to an increased frequency in the application of targeted communication techniques, demonstrating its potential to enhance practical communication skills. Finally, the participating physicians reported that the training had a beneficial impact on their daily clinical practice. This suggests that feedback provided by AI may positively influence health care professionals' perceived competence and confidence.

### Feedback Accuracy

Our results show that AI-generated feedback is mostly accurate, indicating very satisfactory results for a new application of AI. Most participants also found the feedback to be accurate and helpful, supporting the use of AI for real-time evaluation of communication techniques. This aligns with the findings of Imel et al [19], who found that participants generally perceived AI-generated feedback as accurate and helpful for their clinical performance. Furthermore, this confirms prior findings on the effectiveness of AI being comparable to generic experts [45] and extends them by demonstrating that the AI is capable of detecting not only motivational interviewing techniques [19-21] but also communication techniques from Rogers and Linehan. This is particularly noteworthy as these techniques extend beyond the complexity and scope of MI, offering the potential to substantially enhance communication skills of general practitioners in everyday practice. This finding has practical relevance, as reliable AI feedback enables objective assessment of communication skills and enhances communicative competence in clinical settings. Unlike previous work in educational settings, where ChatGPT feedback was evaluated without iterative refinement [46], our study incorporated a process in which AI-generated feedback was modified and refined based on experts´ rating. In line with previous recommendations [47], we trained our AI models on context-specific data to enhance the quality and usability of the feedback.

However, performance varied across techniques, likely due to the low frequency of some techniques (eg, “including past experiences”) in both training and chat data, limiting the AI’s ability to acquire them effectively. A certain error rate with false positive and false negative errors remained. These misclassifications are a common issue [46,48] and may limit training validity and warrant further refinement of the feedback algorithms.

### Increase in the Use of Communication Techniques

A key aim of the study was to assess whether AI-generated feedback encourages more frequent use of communication techniques. Our results show a significant increase between the first and second chat, suggesting that AI-based training may support the correct application of these techniques. These findings are in line with those of Cook et al [49], who reported substantial benefits of virtual patients compared with no intervention. These findings are in line with a recent review [50] on the affordances of AI-based tools, indicating that learners primarily develop their communication skills through prompt feedback and tailored recommendations. Our findings may extend this work by suggesting that real-time AI feedback could serve as a practical approach to enhance communication training. In addition, a recent study by García-Torres et al [51] addressed implementation challenges identified in previous research, such as expanding the application of AI to new communication techniques and clinical domains [51]. While an increase in technique use was observed, causality cannot be confirmed. Further research is needed to evaluate the specific impact of AI feedback in this context.

### Perceived Benefits in Clinical Practice

In addition to quantitative improvements, the majority of the participants reported that AI-based training helped them practice and apply new communication techniques and enhanced their self-perceived ability to recognize depression and anxiety in real clinical interactions. The follow-up survey confirmed these impressions and highlighted perceived long-term benefits. These findings support previous suggestions indicating that AI-based training is not only effective in skill acquisition but also translates into tangible benefits in real-world medical practice [50]. This aligns with the findings of Imel et al [19], who reported that participants expressed a high likelihood of applying the received feedback in practice, underlining the potential of such feedback to foster behavioral change. However, the perceived benefit of AI-based training was found to be influenced by the perceived authenticity of the chatbot. This aligns with prior research, indicating that the effectiveness of virtual training tools depends on their realism and user engagement [51,52]. The importance of developing AI training systems that can simulate real-world interactions as accurately as possible is emphasized by this.

### Implications for Medical Training

This study highlights the potential of AI-powered training to meet the increasing demand for mental health care [53,54] by equipping GPs with key communication techniques. Such tools provide scalable, accessible, and consistent learning opportunities and therefore may represent a valuable addition to future training programs. Compared to traditional training methods, AI-based chatbot training offers several distinct advantages, such as a wide range of application, scalable and flexible access, and allowing learners to practice repeatedly and at their own pace [55]. The real-time feedback is particularly valuable in medical education as it allows learners to immediately correct and refine their skills [56,57], supplementing traditional instruction with individualized, continuous training. Furthermore, AI-generated feedback can be provided immediately, consistently, and without requiring trained human observers [15,17]. While text-based AI cannot fully replicate the complexity, emotional dynamics, or unpredictability of real patient interactions, its availability, repeatability, and standardization make it a valuable supplement for communication training. It can help to maximize learning benefits while mitigating risks such as model bias or overreliance. AI feedback should complement, not replace, expert supervision and be integrated into structured training programs with clear guidance on its interpretation and application [51,52]. In order to ensure the successful integration of technology into the educational environment, educators must adapt curricula and assessment strategies to the evolving technological landscape [58].

### Limitations and Future Research

Despite these promising results, this study presents certain limitations. A primary limitation concerns arises from its exclusive focus on short-term skill acquisition and subjective benefits. Consequently, the results primarily reflect perceived rather than objective or long-term improvements. Additionally, the study incorporated a rather small sample size of 28 physicians from a single site, and participation was voluntary. This may limit the generalizability of the findings and may have introduced self-selection bias, as participants could have been more motivated or interested than nonparticipants. Another relevant issue is the absence of a control group, limiting the ability to draw causal conclusions regarding the impact of AI-generated feedback on the correct use of communication techniques. Finally, the moderate κ values for agreement between the 2 raters of the communication techniques in the pretest likely reflect the complexity of the techniques, whose application can vary even among experts, making uniform assessment difficult. This inherent variability has to be considered when interpreting the results and examined more closely in future studies. Potential risks that also have to be considered especially in the field of medicine include privacy issues, overreliance on automated feedback, model bias, and occasional inaccuracies [59]. It is only if AI feedback is entirely reliable that it can be considered a suitable replacement for a human supervisor. Ethical aspects such as transparency, accountability, and data privacy must also be taken into account.

Future research may benefit from refining AI models to improve feedback reliability and accuracy. Additionally, incorporating longitudinal designs may help to investigate long-term retention of communication skills. Objective outcome measures, such as assessments by patients or recordings of real consultations, could provide a framework for further evaluation of the impact on patient outcomes. Expanding studies to larger, more diverse, multicenter samples may help confirm and generalize these findings. To strengthen causal inference, future research could consider comparisons with a nonintervention control group and randomized controlled trials. This can help clarify the causal contribution of AI-generated feedback to learning new communication techniques.

### Conclusion

In conclusion, this proof-of-concept study highlights the potential of AI-generated feedback in enhancing physicians' communication techniques. The study addressed the research questions by demonstrating that the AI-generated feedback was mostly reliable and was rated as accurate and helpful by the participants. In addition, it has been shown that the AI-supported training resulted in an increased application of specific communication techniques, and that participants reported benefits for their daily clinical practice. These findings suggest that AI-based feedback may represent a valuable addition to medical communications training while also highlighting areas for further research and refinement.

## Appendix

Multimedia Appendix 1 Exemplary feedback on communication techniques used.

Multimedia Appendix 2 Real-time feedback prompts.

Multimedia Appendix 3 Prompts.

Multimedia Appendix 4 Review and evaluation of the AI-generated real-time feedback by experts. AI: artificial intelligence.

## Abbreviations

AI: artificial intelligence
DSM-5: Diagnostic and Statistical Manual of Mental Disorders (Fifth Edition)
GP: general practitioner
LLM: large language model
MI: motivational interviewing
QAP: question-answer pair

## Data Availability

The data that support the findings of this study are available from the corresponding author (LH) upon reasonable request.
