# Supplementary figures and images for: Evaluation of an AI-Based Chatbot Providing Real-Time Feedback in Communication Training for Mental Health Care Professionals: Proof-of-Concept Observational Study
Source: J Med Internet Res. 2025 Nov 28;27:e82818. doi: 10.2196/82818 (PMC12701347; doi:10.2196/82818)

#
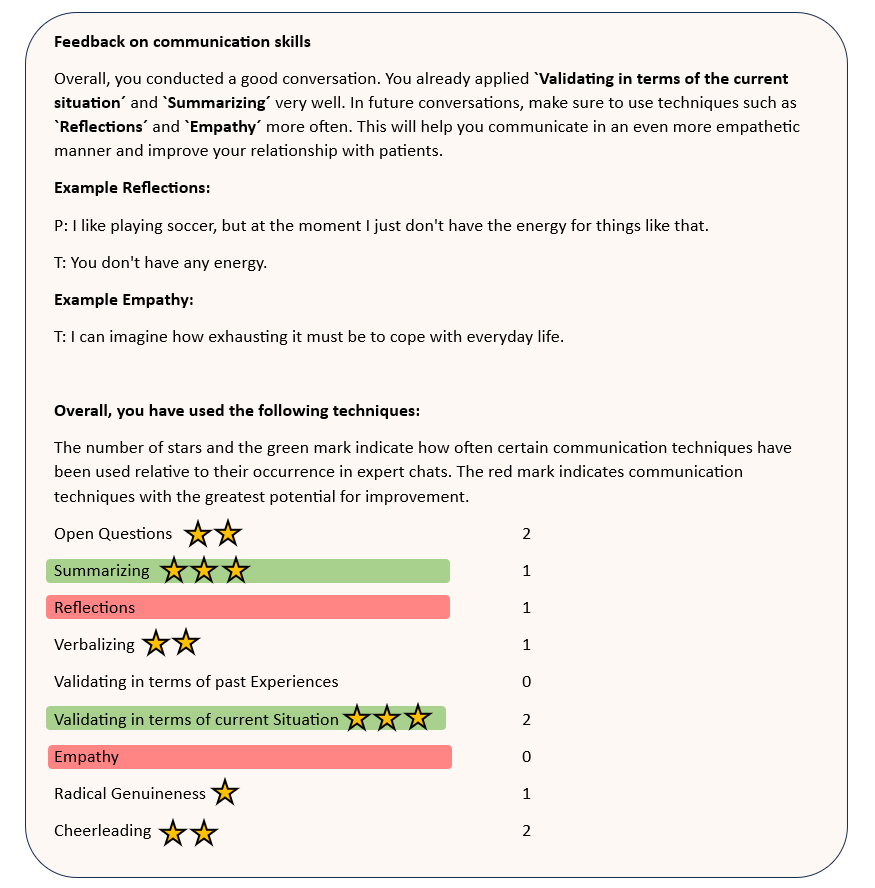
Multimedia Appendix 1: Exemplary feedback on communication techniques used.

Supplement: Multimedia Appendix 1 [file jmir_v27i1e82818_app1.docx]

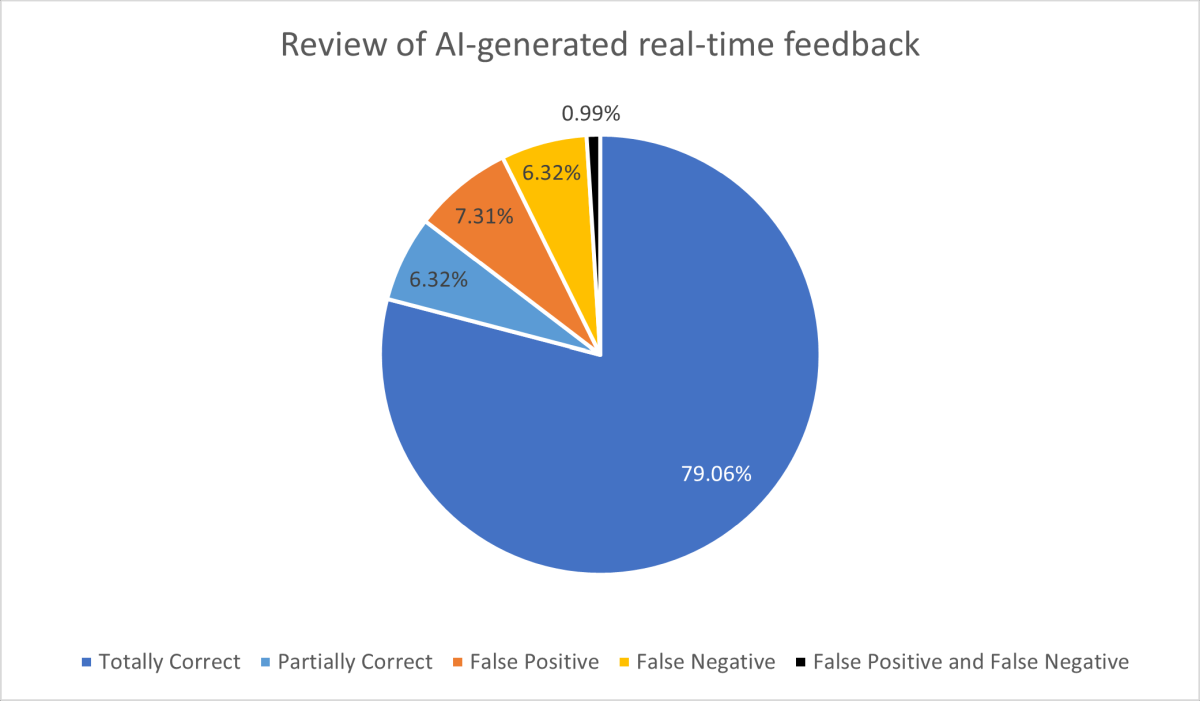

Supplement: Multimedia Appendix 4 [file jmir_v27i1e82818_app4.png]
