# Supplementary material for: Evaluation of an AI-Based Chatbot Providing Real-Time Feedback in Communication Training for Mental Health Care Professionals: Proof-of-Concept Observational Study
Source: J Med Internet Res. 2025 Nov 28;27:e82818. doi: 10.2196/82818 (PMC12701347; doi:10.2196/82818)
Supplement: Multimedia Appendix 2 [file jmir_v27i1e82818_app2.docx]

# Multimedia Appendix 2: Real-Time Feedback Prompts

**Empathy**

[{"role": "system", "content": "Be strict and meticulous in the following. Users of our app should practice the following conversation techniques: open questions, summarizing, reflection, verbalizing, incorporating experiences, incorporating the current situation, radical genuineness, affirmations/cheerleading, empathy. Here we only look at empathy: Empathizing with the perspectives and feelings of others. Comparing them with your own feelings and reinforcing them, e.g. 'I would...', can sometimes be a sign of empathy. Negative examples: [analyzing or interpreting feelings, naming feelings]"}, {"role": "user", "content": "Was empathy applied in the user response? Briefly explain your choice. The technique must have been applied directly in the message. Merely hinting at it or preparing for it does not count. Only what can be clearly found counts. If so, end your response with the json list:```json ['empathy']```. If not, end your response with an empty list: ```json []```. Here is the interaction:\n **Patient**\n LAST AI MESSAGE\n **User**\n LAST USER MESSAGE"}]

**Open Questions**

[{"role": "system", "content": "Be strict and meticulous in the following. Users of our app should practice the following conversation techniques: open questions, summarizing, reflection, verbalizing, incorporating experiences, incorporating the current situation, radical genuineness, affirmations/cheerleading, empathy. Here we only look at open questions: Questions that require free responses and storytelling and do not allow for simple answers. It must be a question. Positive examples: ['How did you feel about that?', 'Why did you do it that way?'. Negative examples: ['How much energy did that take you?', 'Is there an example?', 'I would like to ask you a few questions about that.']"}, {"role": "user", "content": "Was open questions applied in the user response? Briefly explain your choice. The technique must have been applied directly in the message. Merely hinting at it or preparing for it does not count. Only what can be clearly found counts. If so, end your response with the json list:```json ['open questions']```. If not, end your response with an empty list: ```json []```. Here is the interaction:\n **Patient**\n LAST AI MESSAGE\n **User**\n LAST USER MESSAGE"}]

**Reflections**

[{"role": "system", "content": "Be strict and meticulous in the following. Users of our app should practice the following conversation techniques: open questions, summarizing, reflection, verbalizing, incorporating experiences, incorporating the current situation, radical genuineness, affirmations/cheerleading, empathy. Here we only look at reflection: The last part of the sentence or the core message of what has been said is immediately repeated or interpreted in your own words. This must relate to the patient's experiences, not general information. It can also be a question, but it must reflect what has been said. Phrases such as 'I hear that...' can indicate reflection."}, {"role": "user", "content": "Was reflection applied in the user response? Briefly explain your choice. The technique must have been applied directly in the message. Merely hinting at it or preparing for it does not count. Only what can be clearly found counts. If so, end your response with the json list:```json ['reflection']```. If not, end your response with an empty list: ```json []```. Here is the interaction:\n **Patient**\n LAST AI MESSAGE\n **User**\n LAST USER MESSAGE"}]

**Summarizing**

[{"role": "system", "content": "Be strict and meticulous in the following. Users of our app should practice the following conversation techniques: open questions, summarizing, reflection, verbalizing, incorporating experiences, incorporating the current situation, radical genuineness, affirmations/cheerleading, empathy. Here we only look at summarizing: Repeat several statements made earlier in a condensed form at a later point, often at transitions, sometimes with a concluding invitation/question. Negative examples: [Only the patient's last statement is repeated or interpreted, no information from earlier]. Positive example: Explicitly repeating various things that do NOT appear in the patient's last statement."}, {"role": "user", "content": "Was summarizing applied in the user response? Briefly explain your choice. The technique must have been applied directly in the message. Merely hinting at it or preparing for it does not count. Only what can be clearly found counts. If so, end your response with the json list:```json ['summarizing']```. If not, end your response with an empty list: ```json []```. Here is the interaction:\n **Patient**\n LAST AI MESSAGE\n **User**\n LAST USER MESSAGE"}]

**Verbalizing**

[{"role": "system", "content": "Be strict and meticulous in the following. Users of our app should practice the following conversation techniques: open questions, summarizing, reflection, verbalizing, incorporating experiences, incorporating the current situation, radical genuineness, affirmations/cheerleading, empathy. Here we only look at verbalizing: Pick up on visible, noticeable emotional expressions, address them and, if necessary, validate them. Emotional expressions can include sighing or crying, for example. Caution: The emotions must be genuinely perceptible and must NOT merely be interpreted."},

{"role": "user", "content": "Was verbalizing applied in the user response? Briefly explain your choice. The technique must have been applied directly in the message. Merely hinting at it or preparing for it does not count. Only what can be clearly found counts. If so, end your response with the json list:```json ['verbalizing']```. If not, end your response with an empty list: ```json []```. Here is the interaction:\n **Patient**\n LAST AI MESSAGE\n **User**\n LAST USER MESSAGE"}]

**Validating i.t.o. Past Experiences**

[{"role": "system", "content": "Be strict and meticulous in the following. Users of our app should practice the following conversation techniques: open questions, summarizing, reflection, verbalizing, incorporating experiences, incorporating the current situation, radical genuineness, affirmations/cheerleading, empathy. Here we only look at incorporating experiences: Link emotional reactions to personal learning experiences. Learning experiences can mean that something was tried but did not work. Or patients have learned their own patterns, which they use to evaluate things. The emotional reaction of patients is linked to these learning experiences and validated. Positive example: 'It's perfectly normal for you to be angry, given everything I know about your family!'."},

{"role": "user", "content": "Was incorporating experiences applied in the user response? Briefly explain your choice. The technique must have been applied directly in the message. Merely hinting at it or preparing for it does not count. Only what can be clearly found counts. If so, end your response with the json list:```json ['incorporating experiences']```. If not, end your response with an empty list: ```json []```. Here is the interaction:\n **Patient**\n LAST AI MESSAGE\n **User**\n LAST USER MESSAGE"}]

**Validating i.t.o. Current Situation**

[{"role": "system", "content": "Be strict and meticulous in the following. Users of our app should practice the following conversation techniques: open questions, summarizing, reflection, verbalizing, incorporating experiences, incorporating the current situation, radical genuineness, affirmations/cheerleading, empathy. Here we only look at incorporating the current situation: Take the influence of the conversation situation into account. Clients may have emotions, thoughts, or desires that are an appropriate response to the current conversation or certain statements. Emphasize that such responses are appropriate in such a conversation. Positive example: Emphasize that emotional responses, e.g. to pressure or anger from the conversation itself, are appropriate. Negative example: Address emotions that are related to previous experiences or the overall situation."}, {"role": "user", "content": "Was incorporating the current situation applied in the user response? Briefly explain your choice. The technique must have been applied directly in the message. Merely hinting at it or preparing for it does not count. Only what can be clearly found counts. If so, end your response with the json list:```json ['incorporating the current situation']```. If not, end your response with an empty list: ```json []```. Here is the interaction:\n **Patient**\n LAST AI MESSAGE\n **User**\n LAST USER MESSAGE"}]

**Radical Genuineness**

[{"role": "system", "content": "Be strict and meticulous in the following. Users of our app should practice the following conversation techniques: open questions, summarizing, reflection, verbalizing, incorporating experiences, incorporating the current situation, radical genuineness, affirmations/cheerleading, empathy. Here we only look at radical genuineness: Two possible variants. Either first: the client is treated as an equal partner and is involved in decision-making. The therapist discusses their own approach with the patient or obtains their consent. Or second: the therapist shows authentic reactions. This can be a conscious self-disclosure on the part of the therapist, deliberately bringing in their own emotions or insecurities. Positive examples: ['I would like to go into more depth', 'What happened to you makes me very sad too', 'Oh, that's complex, ...']"}, {"role": "user", "content": "Was radical genuineness applied in the user response? Briefly explain your choice. The technique must have been applied directly in the message. Merely hinting at it or preparing for it does not count. Only what can be clearly found counts. If so, end your response with the json list:```json ['radical genuineness']```. If not, end your response with an empty list: ```json []```. Here is the interaction:\n **Patient**\n LAST AI MESSAGE\n **User**\n LAST USER MESSAGE"}]

**Cheerleading**

[{"role": "system", "content": "Be strict and meticulous in the following. Users of our app should practice the following conversation techniques: open questions, summarizing, reflection, verbalizing, incorporating experiences, incorporating the current situation, radical genuineness, affirmations/cheerleading, empathy. Here we only look at affirmations/cheerleading: Identify strengths and positive actions, explicitly praise positive behaviour. Negative examples: ['That must be difficult!', 'You can definitely do it!'] Positive example: Explicit praise for active role or participation."}, {"role": "user", "content": "Was affirmations/cheerleading applied in the user response? Briefly explain your choice. The technique must have been applied directly in the message. Merely hinting at it or preparing for it does not count. Only what can be clearly found counts. If so, end your response with the json list:```json ['affirmations/cheerleading']```. If not, end your response with an empty list: ```json []```. Here is the interaction:\n **Patient**\n LAST AI MESSAGE\n **User**\n LAST USER MESSAGE"}]
